# Factors associated with acute kidney injury recovery in a tertiary hospital in Ghana: a prospective study

**DOI:** 10.11604/pamj.2019.33.236.15507

**Published:** 2019-07-19

**Authors:** Perditer Okyere, Isaac Okyere, Thomas Akuetteh Ndanu, Charlotte Osafo, Bright Amankwaa

**Affiliations:** 1 Department of Medicine, Kwame Nkrumah University of Science and Technology (KNUST) and Komfo Anokye Teaching Hospital, Kumasi, Ghana; 2 Department of Surgery, Kwame Nkrumah University of Science and Technology (KNUST) and Komfo Anokye Teaching Hospital, Kumasi, Ghana; 3 School of Medicine and Dentistry, University of Ghana, Accra, Ghana

**Keywords:** AKI, recovery rate, associated factors

## Abstract

**Introduction:**

Acute kidney injury (AKI) is a challenging problem in developing countries due to late presentation of its victims to health care facilities. Data on the pattern of AKI, its outcome and factors associated with its recovery is scanty in developing countries therefore impeding AKI management. Aim: to study AKI recovery rate and its associated factors.

**Methods:**

An observational study conducted from September 2013 to June 2014 at Korle-Bu Teaching Hospital (KBTH). Participants were adults, admitted with AKI at KBTH. Kidney Disease: Improving Global Outcomes (KDIGO) criteria was used to diagnose and stage AKI.

**Results:**

Mean age (SD) of the participants was 41.9 (± 19.2) years. About a third of the patients (34.6%) were less than 29 years with 30-39 years and 40-60 years constituting 23.0% and 23.6% respectively. Females were in the majority (56.0%). AKI stages I, II and III accounted for 11.0%, 6.8% and 70.7% respectively. Majority, 82.2% of the patients recovered their kidney function. Stage III AKI was significantly associated with decreasing odds of recovery [OR = 0.4, 95%CI = 0.4-2.6, p = 0.002]. In addition, normal blood sodium was associated with recovery from AKI [OR, 95%CI = 2.3, (1.1-5.3), p = 0.043]. Almost half (45.5%) presented with fever whereas 32.5% and 22.5% presented with peripheral oedema and pulmonary oedema respectively.

**Conclusion:**

The study demonstrated high kidney function recovery following AKI. Dominant clinical features were fever, peripheral and pulmonary oedema. Advanced stage was associated with poor recovery whereas normal serum sodium level improves kidney function recovery.

## Introduction

AKI is an important risk factor for chronic kidney disease (CKD), rapid progression to end-stage renal disease (ESRD), development of cardiovascular events (stroke, myocardial infarction), disability, diminished quality of life, high cost of treatment and death [1, 2]. According to Hoste & Schurgers , 2-3 cases of AKI are estimated per 1000 persons [3]. Nash *et al*. estimated that 7% of hospitalized patients at intensive care unit [4] develop AKI, often as a part of multiple organ dysfunctions [5, 6]. AKI patients have nine-time risk of CKD development and two-time risk of premature death than patients without AKI [2]. In developing countries, like Ghana, AKI affects young individuals who are previously well and thus predisposing them to high morbidity, mortality and high cost [7, 8]. AKI is reversible and treatment inexpensive if detected early and the underlying cause managed aggressively [9]. However, at Korle-Bu Teaching Hospital (KBTH) in Ghana, cases of AKI are often referred very late from district hospitals as medical emergencies requiring urgent renal replacement therapy. Patients are often unable to give the exact clinical picture of their illness and this sometimes make it difficult to differentiate AKI from CKD/ESRD. Sonographic evaluation by looking at the cortico-medullary differentiation (CMD) of the kidneys does not help in this differentiation either since poor CMD can be a feature of both AKI and CKD or ESRD. In such late presentations and referrals, recovery from AKI becomes a problem [10].

The prognosis of AKI depends hugely on the underlying aetiology. Knowledge of the factors associated with renal recovery are important. In-depth knowledge of the recovery rate and its associated factors will help to guide decisions concerning treatment and prognosis. The overall aim of this study was to gain insight into the demographics, pattern and outcome of patients with AKI admitted to a tertiary hospital in Ghana. We hope to create awareness on AKI by educating the general public on the demographics, clinical presentations, factors associated with recovery of kidney function and its outcome. More importantly, the findings from this study will enable policy makers; both governmental and non-governmental to make decisions concerning prevention and factors that significantly affect recovery from AKI.

## Methods

**Study design and setting:** this was an observational prospective study carried out at Korle-Bu Teaching Hospital (KBTH). KBTH is a tertiary referral centre located in the capital of Ghana, Accra. The hospital has a 1,600 bed capacity and twelve different departments. Being the largest hospital in Ghana, it receives referrals from all other hospitals in the country. This study was conducted from September 2013 to June 2014.

**Study population and subject recruitment:** any patient who met the Kidney Disease: Improving Global Outcomes (KDIGO) criteria for AKI was eligible for recruitment. In accordance with this guideline, patients were deemed to have AKI if their calculated baseline serum creatinine increase by 26umol/l within 48 hours, an increase in serum creatinine by > 1.5 times the baseline value within 7 days and/or a fall in urine output of <0.5ml/kg/hour for more than 6 hours. Patients with a previous history of chronic kidney disease, patients with ultrasonography evidence of chronic kidney disease (i.e. shrunken kidneys), patients already on chronic haemodialysis therapy and/or patients aged less than 14 years were all excluded from the study. In all, 230 patients were recruited but 39 were lost to follow-up and were therefore excluded from analysis. One hundred and ninety one patients were therefore included in the analysis. Patients were contacted at or after 3 months (ninety days) following diagnosis for a repeat serum creatinine. Patients who had abnormal serum creatinine levels were referred for follow up as chronic kidney disease at the Renal Unit.

**Recovery assessment:** outcome measures were mortality at 30 and 90 days and recovery of renal function at 3 months. For the purpose of this study, full recovery from AKI was considered and was based on the return of a patient's serum creatinine levels to the calculated baseline levels within the KDIGO criteria (absence of AKI) at 3 months [11].

**Ethical consideration:** formal approval was obtained from the Ethical and Protocol Committee of the University of Ghana Medical School. Participation was voluntary and the participants were at liberty to withdraw from the study at any time with no consequence. Patients were fully informed about the nature of the study. Follow up of patients were done within the patient scheduled review period so as to avoid extra burden.

**Data collection:** socio-demographic characteristics including age, gender and clinical presentations were retrieved from participants' clinical records.

**Blood pressure measurement:** blood pressure (BP) measurements were done using the Omron M5-I digital fully automatic blood pressures monitor (Omron, Japan); at least two measurements were taken and averaged. Hypertension was defined if the averaged BP was greater than 140/90mmHg.

**Blood sampling, processing and biochemical assays:** whole blood samples were drawn by venipuncture by competent personnel and collected into both serum separator gel and EDTA tubes, maintained at 0°C - 4°C during delivery to the laboratory. Whole blood was analyzed for haemoglobin concentrations with Sysmex haematology analyzer whereas serum was used for the measurement of creatinine concentration with Cobas C 11 chemistry analyzer.

**Data analysis:** data from patients were analysed using Statistical Package for Social Scientists (SPSS version 20.0). Numeric data were summarized by means and Standard deviation. Categorical data was summarized by frequencies and percentages. Binary logistic regression was used to assess associations between variables with the levels of associations presented as Odds Ratio (OR) and 95%CI. Graphical presentations were provided to highlight the level of differences. Proportions of categorical variables were compared using chi-square test. Significant level was set at α=0.05, thus all statistical tests were declared significant for p-value <0.05.

## Results

**Figure 1:**
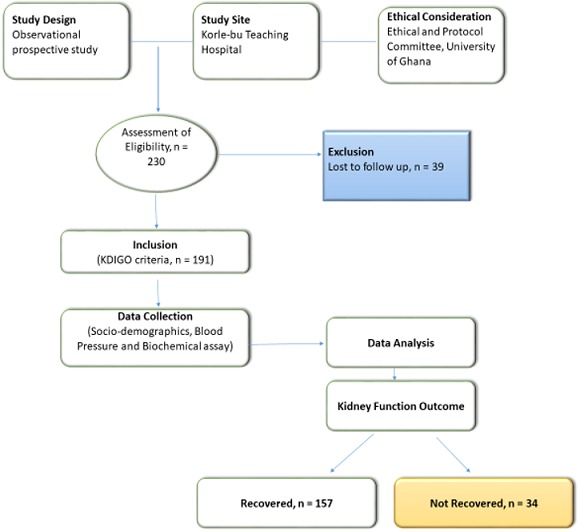
Consort diagram explaining the AKI methodology and kidney function outcome

The mean (±SD) age of the participants was 41.9 (± 19.2) years with 34.6% being ≤ 29 years (Table 1). Females were in the majority (56.0%). 86 (45.0%) of the participants had pre-hypertension whereas 14.1%, and 9.4% had stages I and II hypertension respectively per systolic BP. Anaemia was recorded in 23.0%. In addition, most of the participants had leukocytosis with 92(48.4%) presenting with hyponatraemia (Table 1). From Figure 1, majority (70.7%) of the studied participants were in stage III AKI whereas 11.0% and 6.8% were in stages I and II respectively. Twenty-two of the participants 22(11.5%) had unclassified AKI stage because their biochemical profiles were not available. From Table 2, 45.5% of the AKI participants presented to the hospital with fever. Only 16.8% had diarrhoea. Moreover, 32.5% and 22.5% presented with peripheral and pulmonary oedema respectively. Majority (82.2%) of the studied participants recovered from the AKI attack. However, 17.8% failed to recover (Table 3). The age group 40-60 years and showed non-significant lower odds of recovery [OR, 95%CI = 0.4, (0.2 - 1.1)] compared to those ≤ 29 years. (p = 0.090). Gender was not significantly associated with recovery (p = 0.436). Blood pressure, haemoglobin, WBC, platelets, serum potassium, were not significantly associated with recovery from AKI (p > 0.05). However, stage III AKI was significantly associated with decreasing odds of recovery with reference to stage I [OR = 0.4, 95%CI = 0.4 - 2.6, p = 0.002]. Thus, those with Stage III AKI are 2.5 times less likely of recovery from AKI. In addition, normal blood sodium was associated with increasing odds of recovery from AKI [OR, 95%CI = 2.3, (1.1 - 5.3), p = 0.043]. Those with normal blood sodium are 2.3 times more likely to recover from AKI than those with abnormal blood sodium.

**Table 1:** Socio-demographic, haematologic and biochemical characteristics of study participants

Variables	Frequency	Percentage
	n = 191	(%)
**Age (mean ± SD)**	41.9 ± 19.2	
≤ 29	66	34.6
30 - 39	44	23
40 - 60	45	23.6
>60	36	18.8
**Gender**		
Male	84	44
**Systolic BP(mean ± SD)**	130.7 ± 23.3	
Normal	54	30.4
pre-hypertension	86	45
stage 1	32	16.8
stage 2	15	7.9
**Diastolic BP(mean ± SD)**	80.0 ± 16.9	
Normal	95	49.7
pre-hypertension	51	26.7
stage 1	27	14.1
stage 2	18	9.4
**Serum Creatinine (mean ± SD)**	**783.5(350.8 - 1224.0)**	
**Haemoglobin (mean ± SD)**	9.3 ± 2.9	
Anaemia	44	23
**WBC (mean ± SD)**	13.7(9.1 - 19.7)	
leukocytopenia	7	3.7
leukocytosis	127	66.5
**Platelet (mean ± SD)**	243.0(134.5 - 325.0)	
thrombocytopenia	29	15.2
thrombocytosis	24	12.6
**Serum Sodium (mean ± SD)**	124.7 ± 38.1	
hyponatremia	92	48.4
Hypernatremia	14	74
**Serum potassium(mean ± SD)**	4.5 ± 1.8	
Hypokalaemia	33	17.3
Hyperkalaemia	51	26.7

SD: standard deviation

**Table 2:** Clinical presentation of study participants

Variables	Frequency	Percentage (%)
Fever	87	45.5
Diarrhoea	45	16.8
Rash	32	16.8
Peripheral oedema	62	32.5
Pulmonary oedema	43	22.5

**Table 3:** predictors of recovery from acute kidney injury at Korle-bu Teaching Hospital

Variable	Not Recovered, N = 157(82.2%)	Recovered N = 34(17.8%)	OR(95%CI)	P – value
**Age**				
≤ 29	9(26.5)	57(36.3)	1	
30 - 39	5(14.7)	39(24.8)	1.2(0.4 - 4.0)	0.726
40 - 60	12(35.3)	33(21.0)	0.4(0.2 - 1.1)	0.090
>60	8(23.5)	28(17.8)	0.6(0.2 - 1.6)	0.270
**Gender**				
Male	17(50.0)	67(42.7)	1	
female	17(50.0)	90(57.3)	1.3(0.6 - 2.8)	0.436
**AKI stage**				
Stage I	2(5.9)	19(14.1)	1	
Stage II	4(11.8)	9(6.7)	1.6(0.3 – 14.2)	0.544
Stage III	28(82.4)	107(79.3)	0.4(0.2 – 0.8)	**0.002**
**Systolic BP**				
normal	9(26.4)	49(31.2)	1	
pre-hypertension	13(38.5)	73(46.5)	1.0(0.4 - 2.6)	0.948
stage 1	8(23.5)	24(15.3)	0.6(0.2 - 1.6)	0.275
stage 2	4(11.8)	11(7.0)	0.5(0.1 - 1.9)	0.320
**Diastolic BP**				
normal	17(50.0)	78(49.7)	1	
pre-hypertension	4(11.8)	47(29.9)	2.6(0.8 - 8.1)	0.108
stage 1	7(20.8)	20(12.7)	0.6(0.2 - 1.7)	0.357
stage 2	6(17.6)	12(7.6)	0.4(0.1 - 1.3)	0.143
**Haemoglobin**				
anaemia	8(23.5)	36(22.9)	1	
non-anaemia	26(76.5)	121(77.1)	1.0(0.4 - 2.5)	0.940
**WBC**				
leukocytopenia	2(5.9)	5(3.2)	1	
normal	10(29.4)	47(29.9)	1.9(0.3 - 11.1)	0.486
leukocytosis	22(64.7)	105(66.9)	1.9(0.3 - 10.5)	0.457
**Platelet**				
thrombocytopenia	5(14.7)	24(15.3)	1	
normal	26(76.5)	112(71.3)	0.9(0.3 - 2.6)	0.84
thrombocytosis	3(8.8)	21(13.4)	1.5(0.3 - 6.8)	0.633
**Serum Sodium**				
hyponatremia	22(64.7)	70(44.6)	1	
normal	10(29.4)	74(47.1)	2.3(1.1 - 5.3)	**0.043**
hypernatremia	2(5.9)	12(7.6)	1.9(0.4 - 9.1)	0.429
**Serum potassium**				
hypokalaemia	6(17.6)	27(17.2)	1	
normal	20(58.8)	87(55.4)	1.0(0.4 - 2.7)	0.948
hypernatremia	8(23.5)	43(27.4)	1.2(0.4 - 3.8)	0.765

Data is represented at frequency, n(%), OR = Odds Ratio, CI = confidence interval, WBC = white blood cells

## Discussion

Incidence of AKI is on the rise in developing countries including Ghana [12]. Plethora of factors have been shown to influence outcome of AKI [13]. We therefore sought to find out the associated factors responsible for recovery from AKI in a tertiary Ghanaian hospital. Most of the study participants presented with severe acute kidney injury (AKI stage III) in contrast to a Mexican study in which stage I AKI dominated [13] (Figure 1). Our finding is a cause of concern considering the fact that these severe cases and in most cases late presenters will contribute to the burden of chronic kidney disease in the region where chronic renal replacement therapy remains largely non-affordable. It also reinforces the importance of a management plan that requires patient follow up at 3 months following an episode of AKI. Furthermore, patients who present with severe form of acute kidney injury would require dialysis which at the moment remains very costly for most people in Sub-Saharan Africa [12]. The finding suggests that, acute kidney injury may be a more aggressive disorder in sub-Saharan Africa. This could be due to late presentation to hospital and reliance on clinical criteria for diagnosis, which might only become apparent at an advanced stage. Moreover, most of the patients in this study were referred from hospitals where there is no dialysis facility. This may not be quite different from other countries in sub-Saharan Africa. Results of a survey in Nigeria showed that more than 50% of facilities did not have dialysis capability [14].

**Figure 2:**
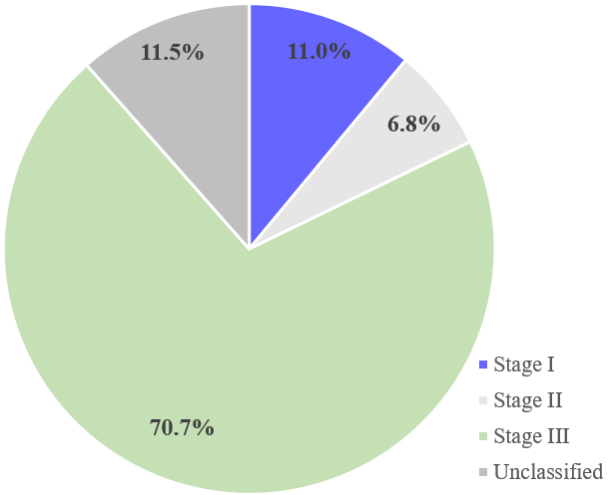
Stages of AKI among the study participants

The study recorded a kidney function recovery rate of 82.2% (Figure 2). This is high compared to previous studies in Nigeria (73.6% to 62.0%) [15, 16]. In Malaysia, Hooi, (1997) also reported recovery rate between 52% and 67% [17]. However, Chaari, 2011 from Tunisia reported a 100% complete recovery, Drakeley 2002 from South Africa reported on 98% complete recovery of kidney function and Arrayhani from Morocco reported recovery rate of 76% [18-20]. This high recovery rate correlates with the fact that most of the patients were young with identifiable and treatable causes (Table 1). Furthermore, patients with more than 3months duration of illness or a previous history of chronic kidney disease or ultrasound evidence of shrunken kidneys were all excluded from the current study. If these patients had been included, the recovery rate might have gone down. Our study found that most of the study participants were in the age ranges 30-39 and 40-60 years (Table 1). This depicts a relatively young population of AKI patients seen at Korle-Bu Teaching Hospital. The age pattern is consistent with a retrospective single-centre study conducted in South-western Ghana in which almost half (40.28%) of AKI participants were in the age range, 40-59 years [21]. Similar age trends have also been reported in Burkina Faso and Morocco [12, 20]. Moreover, in a systematic review, fifteen studies indicated that there was a significant association between young age and the occurrence of AKI [22]. This reiterates the observation that young patients are reported to be a predominant proportion of those presenting with AKI in developing countries and elderly patients reported to be more common in developed countries [22]. In this study, females dominated the AKI participants (Table 1). In Ghana, males are touted to be stoic and reporting to hospitals early is deemed a sign of weakness. This could account for the higher proportion of females and hence higher female AKI participants. Contrary to this gender disparity, a 6-year retrospective study conducted in a Tertiary Health Institution in Northwestern Nigeria found male dominance to females in the ratio, 2:1 [15]. However the finding is in keeping with studies done in, Nigeria, Ethiopia and Sudan [23-25].

In assessing factors associated with recovery from acute kidney injury, the results showed that normal serum sodium concentration significantly increased the likelihood of kidney function recovery (Table 3). Acute kidney injury is often associated with volume overload, electrolyte and acid-base disturbances, particularly hyponatremia which subsequently lead to systemic complications [26]. In the setting of reduced glomerular filtration rate, the ability of the kidney to excrete electrolyte- free water is diminished. To the extent that water intake exceeds this decreased maximal free water excretion, hyponatremia will occur [27]. Moreover, in this study stage III AKI was significantly associated with reduced odds of recovery (Table 3). This agrees with a retrospective cohort analysis of AKI participants in Mexico in which stage III AKI increased mortality by a factor of four (4) [OR=4.5, 95%CI=2.25-8.02, p < 0.001] [13]. According to Ñamendys-Silva, 2016, leukopenia relates to septicaemia and hence more likelihood of recovery failure. Previous studies have also found that patients with kidney disorders have reduced white blood cell counts impaired host defenses [28, 29]. In this study however we did not find any significant association between leucocyte count and recovery from AKI (Table 3). Leucocyte count have been shown to vary significantly from one geographical location to another and therefore might have contributed to the inconsistencies between leucocyte count and AKI recovery. Fever, peripheral oedema and pulmonary oedema were the most common clinical features of the studied AKI patients (Table 2). These findings add to a growing literature regarding the importance of venous congestion manifested as peripheral oedema on renal outcomes [30]. Among a cohort of 12778 critically ill patients in the United States, the presence of AKI on admission was associated with a peripheral oedema [31].

Similar clinical presentations albeit slight variations were reported in a retrospective study conducted among AKI patients in a University Hospital of Kinshasa, Congo in which fever accounted for 80.3% followed closely by jaundice (73.2%) [32]. Unsurprisingly, anaemia was recorded in more than one-fifth of the studied participants in this study (Table 1). In addition, pre-hypertension and hypertension were found in 45.0% and 24.7% among the participants in this study (Table 1). The relationship between AKI and BP elevation is unclear [33]. However, Survivors of AKI were more likely than those without AKI to have elevated BP defined as documented BP>140/90 mmHg in a retrospective cohort study done in Northern California [34]. Blood pressure is considered essential for organ perfusion in that maintaining the optimal blood pressure is an important aspect of preventing acute kidney injury (AKI), especially for vasopressor-dependent patients [34]. Non-availability of chemical analyzers in most hospitals meant that cases of AKI were only picked at peripheral hospital in advanced stage when fluid overload has set in. Patients are therefore likely to be referred to KBTH in advanced stage AKI (i.e. AKI stage III). Whereas some potential factors associated with AKI recovery have been adequately delineated in this study, we could not address partial recovery in participant who may have improved in AKI stage. Moreover, participants who apparently recovered completely from AKI may be prone to developing long-term complications of AKI and a longer follow-up is warranted in future studies.

## Conclusion

The study demonstrates a considerably high recovery rate among the studied AKI participants. This was found to be due to the relatively young nature of AKI diseased patients. Most of the participants were in Stage III AKI. Significant factors found to be associated with recovery from AKI were serum sodium levels and Stage III AKI; whereas normal sodium levels increase odds of recovery, stage III AKI suggests a lesser chance of recovery. Dominant clinical features found in the participants were fever, peripheral and pulmonary oedema. In addition, anaemia and hypertension were important co-morbidities found among the studied participants. Further studies which employ robust study design involving longer follow-ups with recruitment from peripheral health centres are warranted to fully understand the mechanism behind these associations.

### What is known about this topic

- Incidence of acute kidney injury is considerably high in Ghana and other under-resourced countries;
- Acute Kidney Injury is a major risk factor of chronic kidney disease, morbidity and mortality;
- Acute Kidney Injury is presented at later stages in Ghana which potentially affects recovery.

### What this study adds

- The study reports high recovery rate among acute kidney injury patients at Korle-bu Teaching Hospital due probably to the young nature of such patients;
- Normal blood sodium levels and stage III acute kidney injury affects recovery;
- Fever, peripheral oedema and pulmonary oedema are major clinical features associated with acute kidney injury.

## Competing interests

The authors declare no competing interest.
